# Kypho-IORT - a novel approach of intraoperative radiotherapy during kyphoplasty for vertebral metastases

**DOI:** 10.1186/1748-717X-5-11

**Published:** 2010-02-11

**Authors:** Frederik Wenz, Frank Schneider, Christian Neumaier, Uta Kraus-Tiefenbacher, Tina Reis, René Schmidt, Udo Obertacke

**Affiliations:** 1 Department of Radiation Oncology, University Medical Centre Mannheim, Heidelberg University, Mannheim, Germany; 2 Department for Orthopaedics and Trauma Surgery, University Medical Centre Mannheim, Heidelberg University, Mannheim, Germany

## Abstract

**Background:**

Instable and painful vertebral metastases in patients with progressive visceral metastases present a common therapeutic dilemma. We developed a novel approach to deliver intraoperative radiotherapy (IORT) during kyphoplasty and report the first treated case.

**Methods/Results:**

60 year old patient with metastasizing breast cancer under chemotherapy presented with a newly diagnosed painful metastasis in the 12^th ^thoracic vertebra. Under general anaesthesia, a bipedicular approach into the vertebra was chosen with insertion of specially designed metallic sleeves to guide the electron drift tube of the miniature X-ray generator (INTRABEAM, Carl Zeiss Surgical, Oberkochen, Germany). This was inserted with a novel sheet designed for this approach protecting the drift tube. A radiation dose of 8 Gy in 5 mm distance (50 kV X-rays) was delivered. The kyphoplasty balloons (KyphX, Kyphon Inc, Sunnyvale) were inflated after IORT and polymethylmethacrylate cement was injected. The whole procedure lasted less than 90 minutes.

**Conclusion:**

In conclusion, this novel, minimally invasive procedure can be performed in standard operating rooms and may become a valuable option for patients with vertebral metastases providing immediate stability and local control. A phase I/II study is under way to establish the optimal dose prescription.

## Background

It is a common therapeutic dilemma in the treatment of advanced stage cancer that progressive visceral metastases and instable and painful bone metastases are present simultaneously and require urgent treatment. However, due to potentiated toxicity, simultaneous treatment with full dose chemotherapy and fractionated radiotherapy is rarely possible. In addition, instability of the vertebral column may require prolonged periods of bed rest. We have therefore developed a novel approach to deliver intraoperative radiotherapy (IORT) during kyphoplasty in order to regain immediate stability, sterilize the metastasis and continue with chemotherapy without a delay of several weeks. Here we report about the first use of this novel approach (Kypho-IORT).

## Results

**Figure 1:**
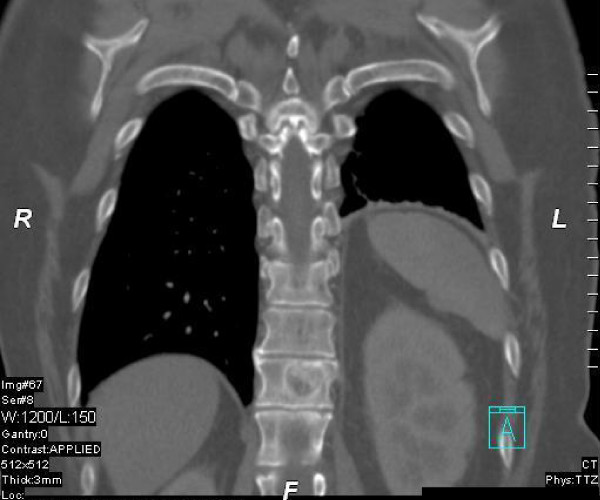
**Coronal CT scan of the vertebral metastasis**.

A 60 year old patient with metastasizing breast cancer to the lung and mediastinal lymph nodes under docetaxel chemotherapy (75 mg/sqm q21d for 3 cycles) presented with a newly diagnosed painful metastasis in the 12^th ^thoracic vertebra. There was an increased risk for a pathologic fracture due to the extent of the vertebral metastasis (see figure 1). The initial diagnosis of a receptor-positive lobular-invasive breast cancer (pT2 pN1a G2 her2neu FISH negative) in the left upper-outer quadrant was made 3 years ago. The patient received initially breast conserving surgery, axillary dissection, adjuvant chemotherapy (6 × FEC) and whole breast radiotherapy with boost (50 + 16 Gy) followed by endocrine therapy using letrozole. Visceral metastases were diagnosed three months ago during routine follow-up and palliative chemotherapy was initiated.

**Figure 2:**
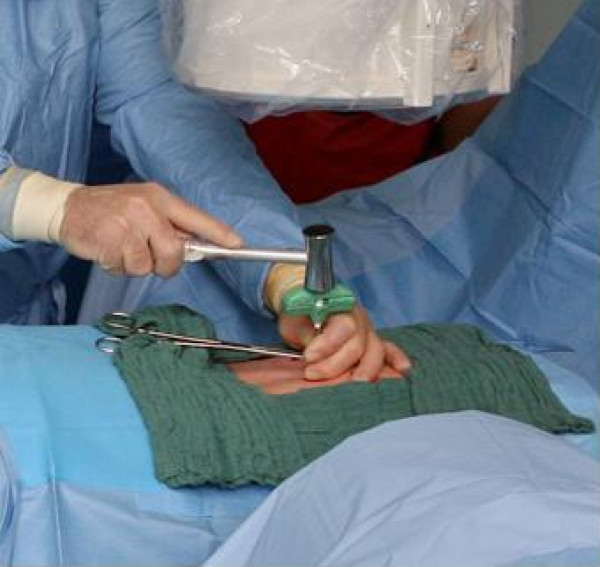
**Specially designed guiding sleeves were inserted using a bipendicular approach**.

**Figure 3:**
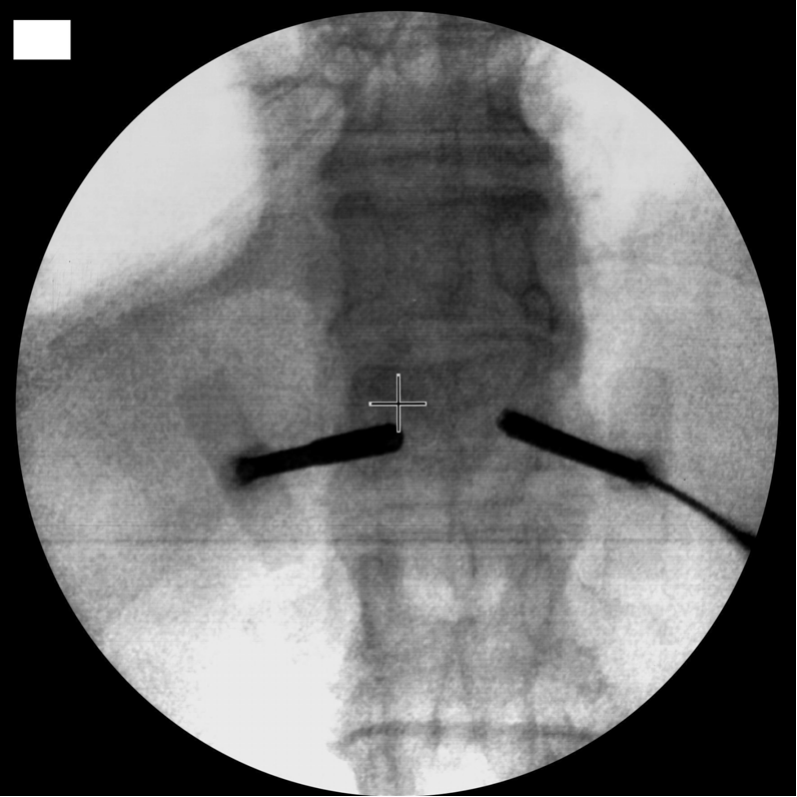
**X-ray control of guiding sleeve position**.

**Figure 4:**
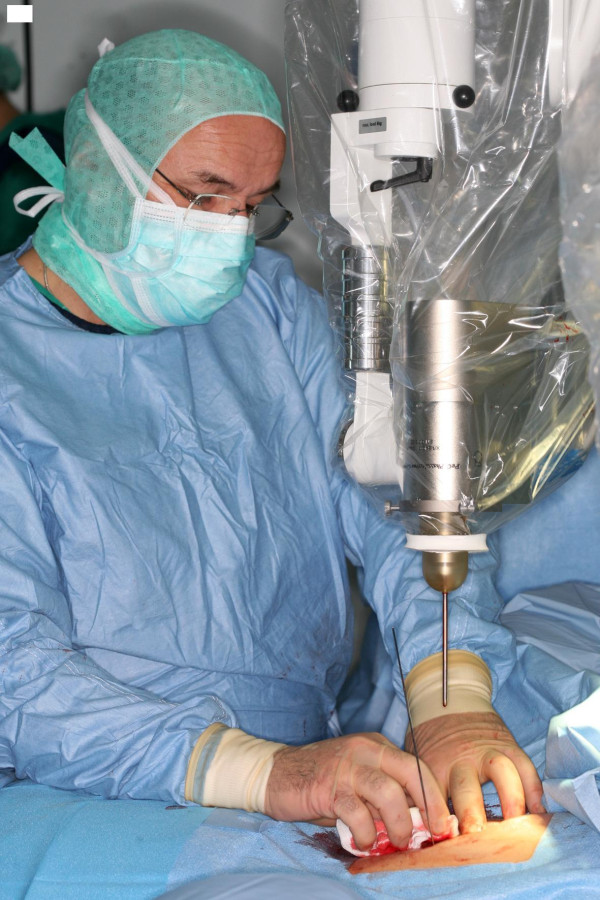
**The INTRABEAM system is inserted into the guiding sleeves while the drift tube is protected by a novel sheet**.

The kyphoplasty itself was performed according to the standard procedure with some minor modifications. In short, under general anaesthesia and after sterile preparation of the patient, a bipedicular approach was chosen (see figure 2). Specially designed metallic sleeves (5 mm diameter, 6 cm length) were inserted (see figure 3) to guide the electron drift tube (3.2 mm diameter) of the miniature X-ray generator (INTRABEAM, Carl Zeiss Surgical, Oberkochen, Germany). After verification of the position of the guiding sleeves using biplanar X-ray, the INTRABEAM system was inserted with a novel sheet (diameter 4.2 mm) designed for this approach protecting the drift tube (see figure 4 + 5). Special care was taken to avoid sheer and bending stress on the drift tube and the sheet. A radiation dose of 8 Gy in 5 mm distance (50 kV X-rays, corresponding to 27 Gy at 2 mm distance from surface of the radiation source) was delivered during about 90 seconds to the center of the metastasis. Examples of dose distributions depending on the position of the radiation source can be seen in figures 6 + 7. Afterwards, the INTRABEAM system was removed. The kyphoplasty balloons (KyphX, Kyphon Inc, Sunnyvale) were inflated with 300 PSI on either side and 2 × 4 ml polymethylmethacrylate (PMMA) cement were injected (KyphX, Kyphon Inc, Sunnyvale, see figure 8). The guiding sleeves were extracted and skin closure was done as usual. The whole procedure lasted less than 90 minutes. The patient was pain free on the day following the procedure and there was no visible radiation induced skin reaction.

**Figure 5:**
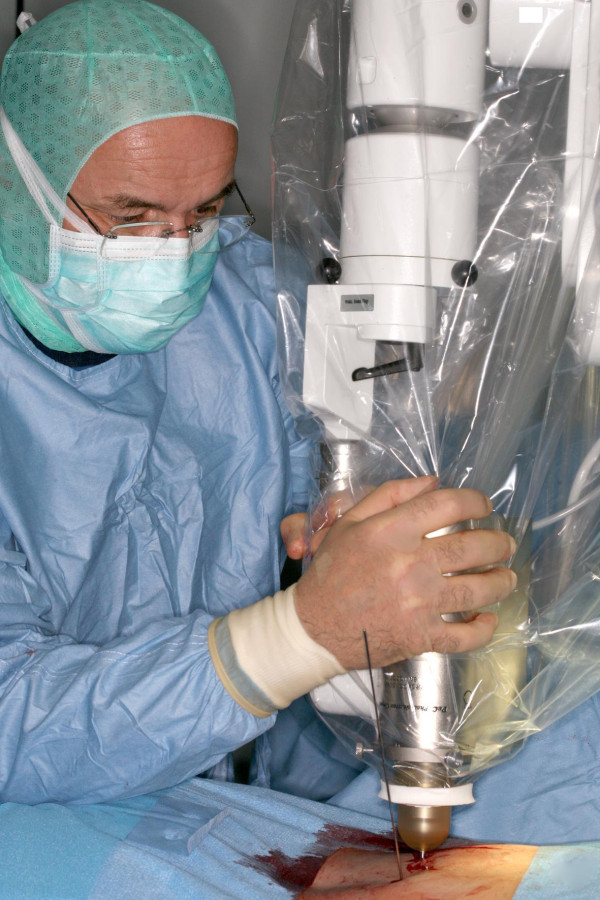
**The INTRABEAM system in treatment position**.

**Figure 6:**
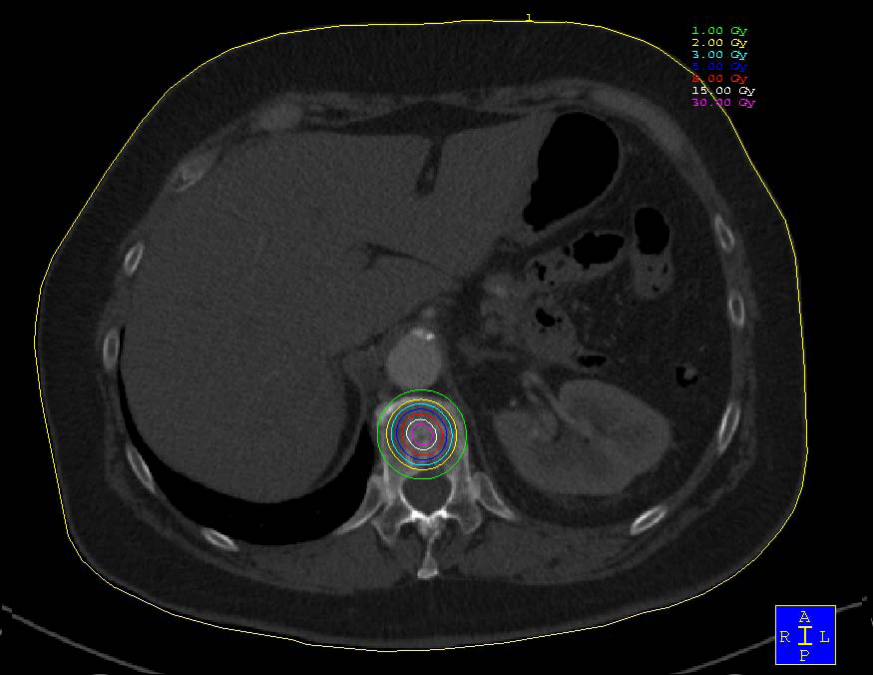
**Dose distribution for localization in the center of the vertebra**. Please note that the spinal cord is touched by the 1 Gy isodose.

**Figure 7:**
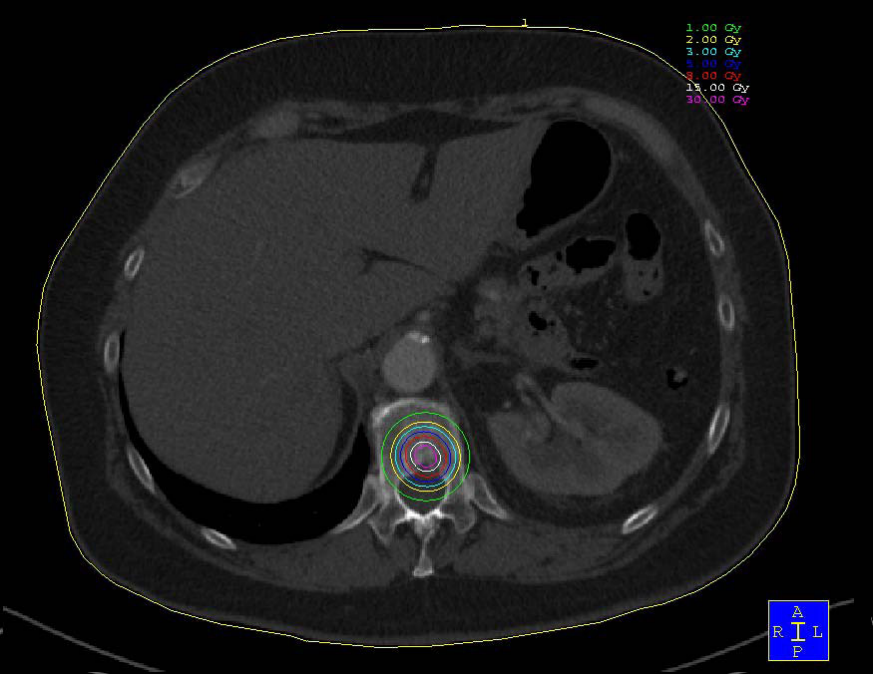
**The 8 Gy isodose reaches the spinal cord after placement of the radiation source in the dorsal part of the vertebra**.

**Figure 8:**
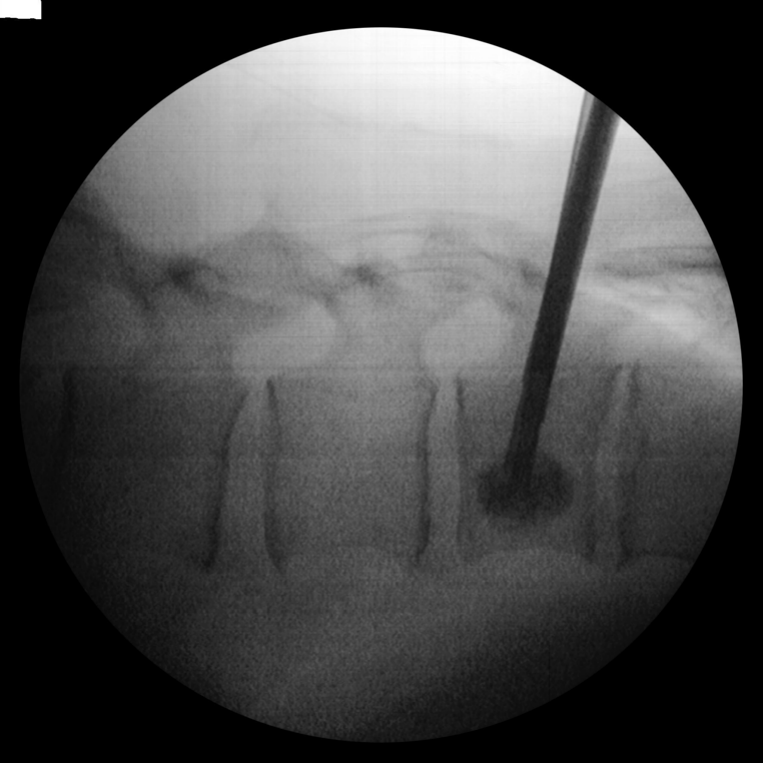
**PMMA cement is injected after inflation of the kyphoplasty balloons to stabilize the vertebra**.

## Discussion

Percutaneous kyphoplasty and vertebroplasty are increasingly used [1-5]. It is a valuable treatment option for patients with painful and instable metastases to the vertebral column although its value for osteoporotic fractures has been recently questioned [6,7]. Now it is possible to combine this procedure with the delivery of a high dose of intraoperative radiotherapy (IORT) providing immediate stability, pain relief and sterilization of the metastasis. Technically, vertebrae below the level of thoracic vertebra 3 are suitable for the Kypho-IORT approach when the metastasis is predominantly located in the vertebral body. A limited destruction of the dorsal corticalis is not a contraindication, however, special attention should be paid to avoid leakage of the PMMA into the spinal canal ("egg-shell technique"). Whether metastases in the pedicle can be approached with this technique remains to be determined based on the dose to the spinal cord and the stability of the positioning of the IORT device. External beam radiotherapy or radiosurgery [8,9] even when combined with bisphosphonates do provide improved structural stability only after a prolonged period of time and the hazards of open radionuclides are avoided with this X-ray based approach [10]. A detailed discussion about different fractionation schedules for the treatment of spinal cord metastases can be found in a recent review [11]. This new application broadens the potential applications of the Intrabeam system, which is up to now mainly used for IORT for breast cancer [12-14], and brain tumors [15]. Because long term experiences with this approach are not available at present, a phase I/II study is ongoing to establish the optimal dose presciption to provide local control.

## Conclusion

IORT with low energy X-rays can be performed during kyphoplasty in standard operating rooms without the necessity of excessive radiation protection measures. As survival times of patients with many types of advanced cancer increase, the demand for this novel approach will potentially increase in the future.

## Competing interests

Carl Zeiss Surgical Oberkochen supports radiobiological research at University Medical Centre Mannheim.

## Authors' contributions

FW, UO, FS: idea and concept; FW, FS, UKT, RS, UO: design and development; CN, UKT, TR, RS: patient selection and care; FW, CN, TR: writing of manuscript; FW, FS, CN, UKT, TR, RS; UO: final revision of manuscript. All authors have read and approved the final manuscript.
